# Multi-alternative decision-making with non-stationary inputs

**DOI:** 10.1098/rsos.160376

**Published:** 2016-08-24

**Authors:** Luana F. Nunes, Kevin Gurney

**Affiliations:** Department of Psychology, University of Sheffield, Sheffield S10 2TP, UK

**Keywords:** MSPRT, decision-making, non-stationary, multi-alternative, basal ganglia

## Abstract

One of the most widely implemented models for multi-alternative decision-making is the multihypothesis sequential probability ratio test (MSPRT). It is asymptotically optimal, straightforward to implement, and has found application in modelling biological decision-making. However, the MSPRT is limited in application to discrete (‘trial-based’), non-time-varying scenarios. By contrast, real world situations will be continuous and entail stimulus non-stationarity. In these circumstances, decision-making mechanisms (like the MSPRT) which work by accumulating evidence, must be able to discard outdated evidence which becomes progressively irrelevant. To address this issue, we introduce a new decision mechanism by augmenting the MSPRT with a rectangular integration window and a transparent decision boundary. This allows selection and de-selection of options as their evidence changes dynamically. Performance was enhanced by adapting the window size to problem difficulty. Further, we present an alternative windowing method which exponentially decays evidence and does not significantly degrade performance, while greatly reducing the memory resources necessary. The methods presented have proven successful at allowing for the MSPRT algorithm to function in a non-stationary environment.

## 1. Introduction

In our daily lives, we are continually faced with the problem of ‘deciding what to do next’ based on a plethora of sensory information and our internal cognitive, and homeostatic state. This process of *decision-making* continues to attract substantial interest in the computational modelling literature (for reviews, see [1,2]). Mechanistically, many of these models assume that noisy sensory streams provide samples of ‘evidence’ for each of several alternatives, and that these evidence samples are accumulated in some way until a reference criterion of distinguishability is reached (defined by one or more thresholds). This is an idea that meshes well with neuroscientific evidence showing ‘evidence accumulation’ in brain areas involved in decision-making [3–5]. Such decision-making processes are also subject to a speed–accuracy trade-off which accords well with psychological studies of reaction times and error rates [1]. Thus, by manipulating the threshold(s), more or less evidence may be allowed to accumulate before the decision is made. Here, longer accumulation (‘reaction’) times lead to greater accuracy or lower error rates.

Most previous studies of decision-making have focused on the choice between two alternatives [6–9]. In this regard, it has been shown that one mechanism in particular—the *sequential probability ratio test* (SPRT)—is optimal in that it takes the least number of evidence samples to reach a decision for a given error rate [10]. Recently, however, there has been a growing interest in understanding the processes involved in multi-alternative decision-making (more than two options) [5,11] and extending models originally developed for binary decision to multi-alternative computation [12–14].

Perhaps, one of the most widely implemented models for multi-alternative decision-making is the *multihypothesis sequential probability ratio test* (MSPRT) [12,15–17]. While this reduces to SPRT for two alternatives, it fails, in general, to reach similar optimality criteria. Thus, MSPRT can be shown only to achieve what is known as ‘asymptotic optimality’ in that it minimizes mean response time as the error rate tends to zero. Even though optimality is not achieved if error rates are not negligible, the performance of MSPRT in this case is still better than some of its competitor mechanisms [13].

Further, the MSPRT has also been mapped onto a neurobiological substrate associated with decision-making, namely—the basal ganglia [18,19]. The latter are a set of evolutionarily old, subcortical nuclei which are believed to play a critical role in action selection and decision-making [20,21]. The fact that the MSPRT is so closely mapped to the basal ganglia anatomy and that this mapping requires very specific anatomical and physiological aspects to be satisfied supports the view that these nuclei may be at least approximating the decision algorithm under certain circumstances.

However, irrespective of mechanism studied, or the number of choices involved, most work on decision-making thus far has assumed a discrete *trial-based* paradigm in which evidence is accumulated from streams with stationary statistics, a decision reached, and then all state variables are reset. Usually, in trial-based schemes, the decision is made when the threshold criterion is reached but, in the *interrogation paradigm* [1], a decision is forced after a predetermined time period. In either case, statistical stationarity and mechanism reset is assumed before the next trial period.

Recently, the ethological validity of these assumptions has been questioned by considering non-stationary stimuli [22,23]. However, these studies still assume trial-based schemes, and also use the leaky competing accumulator decision mechanism (rather than MSPRT). In a fully ethological situation, we expect that, as well as stimulus non-stationarity, there will be no clearly delineated ‘trials’, implying no obvious way of resetting the decision mechanism, and thereby *de-selecting* the current choice.

The question we address here therefore is: how can a decision mechanism continually select *and* de-select actions or choices governed by multiple streams of noisy sensory information? In a trial-based, reset-endowed scheme, ‘old’ evidence from the previous trial is explicitly discarded (at reset) before a new decision is made. In the proposed paradigm, we have to implement an automatic, dynamic mechanism for ‘forgetting’ old evidence. We take as our starting point the MSPRT because we wish to allow subsequent contact with possible basal ganglia implementation, and benefit from the potential for approximate optimality offered by this mechanism.

## 2. Material and methods

We describe here our starting point—the MSPRT algorithm, conceived as a trial-based process, and interpreted in a form suitable for mapping to basal ganglia operation.

Consider *N* streams or *channels* of sensory information *x*_*i*_(*t*), which are sampled at discrete times *t*, from stationary random variables (fixed mean and standard deviation). We now form a hypothesis *H*_*i*_, for each channel *i*, which is that *x*_*i*_(*t*) is sampled from a distribution *f*^+^ with mean and standard deviation *μ*^+^, *σ*, while all the others are sampled from counterparts *f*^−^ with parameters *μ*^−^, *σ*, where *μ*^+^>*μ*^−^. If *X*(*T*) is the entirety of sensory information up to some time *T*, we wish to find the posterior probability *P*(*H*_*i*_ | *X*(*T*)). It can be shown [18,19] that the required input to a Bayesian decision mechanism for determining these are the ‘evidence’ samples formed from the per-sample log-likelihood ratios

2.1 $$LR_{i}(t)=\ln\frac{f^{+}(x_{i}(t))}{f^{-}(x_{i}(t))}.$$

We now specialize to Gaussian random variables as we do so in the rest of the paper. In this instance, equation (2.1) becomes

2.2 $$LR_{i}(t)=gx_{i}(t)-g\frac{\mu^{+}+\mu^{-}}{2},$$

where

2.3 $$g=\frac{\mu^{+}-\mu^{-}}{\sigma^{2}}.$$

It transpires that channel-independent terms do not affect the posterior, and so we may ignore the second term in (2.2) and use *LR*_*i*_(*t*)=*gx*_*i*_(*t*). That is, we can take as our evidence samples the original sensory samples themselves, *x*_*i*_(*t*), interpreting *g* as an overall ‘gain’ factor which depends on problem difficulty (difference between means and noise). This algebraic reduction is a special case applicable only to Gaussian random variables; other distributions yield more complex expression [24]. However, given Gaussian noise, the MSPRT has the interpretation of effectively finding the input channel with the largest mean of its stimulus samples *x*_*i*_(*t*); an interpretation we will use henceforth. The accumulated evidence, *y*_*i*_(*T*), up to time *T*, is then just

2.4 $$y_{i}(T)=g\sum_{t=1}^{T}x_{i}(t).$$

Using Bayes theorem, it may now be shown [18,19] that the log of the posterior $L_{i}(T)\equiv \ln P(H_{i}\;|\;X(T))$ is given by

2.5 $$\begin{array}{rl} L_{i}(T) & =y_{i}(T)-Z_{i}(T)\\ \;\text{with}\;\;Z_{i}(T) & =\ln\left(\sum_{k=1}^{N}\exp(y_{k}(T))\right). \end{array}\}$$

A decision is reached when *L*_*i*_(*T*) crosses a threshold *θ* from below. However, the basal ganglia work by release of inhibition from their target structures in the brain [25]; that is ‘winning’ channels are associated with a decrease in signal values. Thus, if we wish to interpret the output of the MSPRT as the output of the basal ganglia, we need to consider not *L*_*i*_(*T*) but ${\bar{L}}_{i}(T)\equiv -L_{i}(T)$, so that

2.6 $${\bar{L}}_{i}(T)=-y_{i}(T)+Z(T).$$

Now, a decision is reached when ${\bar{L}}_{i}(T)$ crosses the threshold from above [18], which is the scheme we adopt here.

The term *Z*_*i*_(*T*) captures the interaction between channels; for a given amount of evidence *y*_*i*_(*T*) for channel *i*, increasing evidence for other channels increases *Z*_*i*_(*T*), thereby tending to prevent a decision. We therefore refer to *Z*_*i*_(*T*) as the *conflict* or *competition* term.

## 3. Results

### 3.1. MSPRT-based mechanism with windowed sampling performs robust decision-making in non-stationary environments

**Figure 1.**
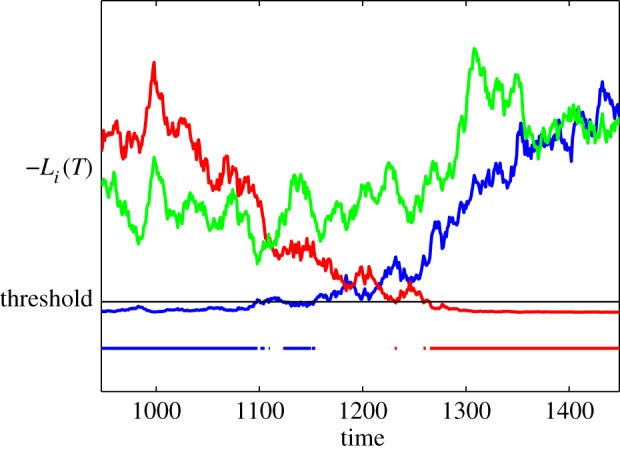
Decision-making with a transparent boundary. There are three competing channels with their outputs, ${\bar{L}}_{i}(T),\;i=1,2,3$, shown in red, green and blue; see equation (2.6). These signals cross the threshold from above and below, with threshold crossing from above resulting in a decision. Decision states are shown in the horizontal lines at the bottom, with the colour indicating the selected channel at any time. Note that not all times are associated with a decision. The signals are representative of those in a trial of figure 4 for the fixed rectangular window (700 ms) in epoch 2, where Δ*μ*=*μ*^+^−*μ*^−^=1 and *σ*=0.3. For this example, the threshold was increased from 0.05 to 0.3 to elicit more selection and better illustrate the switch of selection from one option to another.

In trial-based MSPRT with a single decision, when the threshold is reached, a decision is made, integration stops, and evidence integrators are reset to zero. This is reflected in the decision boundary being *absorbing*—no further processing takes place after a threshold crossing. By contrast, in a temporally extended, non-stationary situation, we require multiple decisions to be made with options which were once selected, being subsequently de-selected in favour of others. This demands that the decision boundary is now *transparent*—the trajectory described by an option may recross the boundary in the opposite direction from that which caused its selection. In addition, subsequent decisions are allowed for any option by threshold crossing for selection. An example of this kind of decision-making is shown in figure 1; between a selection and subsequent de-selection event (threshold crossing) of a channel, it is deemed to be in a‘selected state’. Henceforth, therefore, we assume a transparent decision boundary, unless otherwise stated.

Interestingly, not all decision mechanisms are capable of using a transparent boundary. To do so requires that the expectation value of the dynamic variable which has to reach threshold (posterior probability for MSPRT) can have its direction reversed. This may occur via competitive processes, as are present in the drift diffusion model [7] , or a decay process, as in the leaky competing accumulator (LCA) [26]. However, the relevant variables in the race model [27] are monotonically increasing and so this model does not allow de-selection with a transparent boundary.

To see that the MSPRT is a candidate for non-stationary decision-making, suppose that channel *j*, having been selected at time *T*_*j*_, has no further evidence accumulated after *T*_0_>*T*_*j*_. Then for *T*>*T*_0_, using (2.6), ${\bar{L}}_{j}(T)=-y_{j}(T_{0})+Z(T)$. This is monotonic increasing in *T* because the first term is now fixed and the ‘competition term’, *Z*(*T*) is monotonic. Thus, eventually, ${\bar{L}}_{j}(T)$ recrosses the threshold from below and, once a channel has no more evidence accumulated, this now-outdated evidence will effectively be gradually discarded, resulting in the channel being de-selected.

Now consider channel *i* which is the next to be selected. The time to decision for *i* depends, not only on the rate of increase of evidence *y*_*i*_(*T*), but also on the competition term *Z*(*T*). But *Z*(*T*) includes the evidence accumulated from all the previous ‘experience’ of the decision mechanism, and therefore represents an ever increasing impediment to threshold crossing by *i*, as this experience grows. It is as if the inability to ‘forget’ old information is causing subsequent decisions to be progressively harder.

One way of alleviating this problem is to force the ‘forgetting’ of previous evidence using a temporal *sampling window* and, conceptually, the simplest window form is rectangular. This is equivalent to defining the accumulated evidence as a convolution of the evidence samples and a rectangular function *w*(*t*) with non-zero duration *T*_*w*_. Thus, dropping the channel suffix for simplicity,

3.1 $$\begin{array}{rl} y(T) & =(x\times w)[t] \end{array}$$

3.2 $$\begin{array}{rl} & =\sum_{t=1}^{\infty }x(t)w(T-t), \end{array}$$

where

3.3 $$w(t)=\left\{ \begin{array}{ll} 0, & \text{if }t<0,\\ g, & 0\leq t\leq T_{w}\\ 0, & t>T_{w} \end{array}\right.$$

and *g* is the gain defined in (2.3). Using these limits on non-zero *w*(*t*), we have

3.4 $$y(T)=\sum_{t=T-T_{w}}^{T}g\times x(t).$$

The decision mechanism now incorporates evidence from a fixed number of samples only over this window. Recall that convolution may be thought of as multiplying one function by another which ‘slides’ over the first. Here, we imagine the rectangular window sliding over the data. Note that, if *T*_*w*_=*T*, and *x*(*t*) is stationary, this new mechanism reduces to the normal MSPRT.

In order to test the effectiveness of the moving window method with non-stationary inputs, we compared the results of the original MSPRT (with no windowing) to variations with two fixed windows of short (300 ms) and long (700 ms) duration. We used three channels, with input means *μ*_1_(*t*),*μ*_2_(*t*),*μ*_3_(*t*) and, as described in Material and methods, these were defined by two hypothesis testing means *μ*^+^,*μ*^−^, with *μ*^+^>*μ*^−^. There were two epochs defined according to

3.5 $$\begin{array}{rl} & \text{Epoch 1}\;0\leq t<1000:\;\mu_{1}(t)=\mu^{+},\;\mu_{2}(t)=\mu_{3}(t)=\mu^{-}\\ \;\text{and}\; & \text{Epoch 2}\;1000\leq t<2000:\;\mu_{1}(t)=\mu_{3}(t)=\mu^{-},\;\mu_{2}(t)=\mu^{+}. \end{array}\}$$

The standard deviation of the inputs, *σ*, was fixed throughout this experiment for both channels at 0.33. We explored performance under different difficulty of decision by varying Δ*μ*=*μ*^+^−*μ*^−^ over the closed interval [0.15,1.5] sampling with an increment 0.15.

We also investigated the effect of the decision threshold *θ*, whose variation could show features not apparent in the case of stationary inputs. To fix the threshold range, we note that, at the start of the experiment, the evidence for each channel will be zero. Substituting *y*_*i*_=0 into (2.6) for two channels, we have ${\bar{L}}_{i}(T_{w})=\ln(\exp(0)+\exp(0))=\ln(2)\approx 0.693$. There is clearly no point having a threshold greater than that associated with no evidence, and so this places an upper bound on *θ*; we refer to this as the *zero-evidence limit*. We also used an order of magnitude range, so that *θ* was varied over the closed interval [0.05 0.5] sampling at intervals of 0.05.

**Figure 2.**
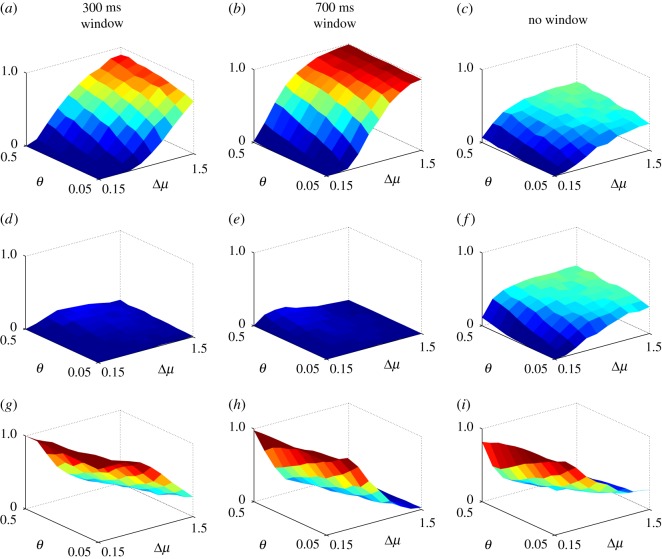
Performance of windowed and non-windowed MSPRT as a function of decision difficulty (defined by Δ*μ*=*μ*^+^−*μ*^−^; see text) and threshold *θ*. The input protocol is that in equation (3.5). Panels (*a*–*c*) show the probability *P*, (fraction of 1000 experiments) of performing a *correct* decision at the end of the trial for each of the cases: 300 ms window, 700 ms and no window. Panels (*d*–*f*) show the corresponding probabilities of performing an *incorrect* decision. Panels (*g*–*i*) show the probabilities for reaching no decision at the end of the trial.

We evaluated performance by examining the outcome at the end of epoch 2, because this represented a typical decision in a non-stationary environment, where the evidence may contain irrelevant contributions from a previous epoch. The results, averaged over 1000 experiments (each with a different instance of the stochastic input), and at each combination of Δ*μ* and *θ*, are summarized in figure 2. A ‘correct decision’ was one in which the channel with the larger input was selected at the end of epoch 2; that is, its negative log-posterior, ${\bar{L}}_{i}$, was less than *θ*. The other possible outcomes were: ‘incorrect decision’ (a channel with the smaller input was selected), ‘no decision’ (no channel had ${\bar{L}}_{i}<\theta$) and ‘multiple selection’ (more than one channel had ${\bar{L}}_{i}<\theta$, this was omitted because no ‘multiple selection’ was observed for these sets of parameters).

Figure 2*a*–*c* shows the probability *P*, of obtaining the correct outcome (fraction of the 1000 runs). The non-windowed (original) MSPRT performs at roughly chance levels for most of the parameter range. This is because the decision mechanism is being called on to work on selecting channel 2 in epoch 2, but is ‘contaminated’ with erroneous evidence which supported channel 1 in the first epoch. By contrast, for both small and large window sizes, there is a threshold range which allows better performance than the non-windowed MSPRT. In particular, the 700 ms window shows an almost perfect performance for a significant range of problem difficulty and threshold. Thus, the ability to ‘forget’ prior evidence can be advantageous.

Furthermore, the 300 ms window benefits from somewhat higher thresholds for low problem difficulty (large Δ*μ*). As the increase in threshold is moderate, it does not result in a significant increase in incorrect selection. The benefit of larger thresholds may seem counter-intuitive because, for the un-windowed MSPRT, smaller thresholds require more evidence to reach, and are associated with lower error-rates. However, with the windowed variants, if the threshold is too low, the time required to reach it may exceed the window time, and somewhat higher thresholds can force selection (albeit not always correct). In addition, reporting the correct decision at the *end* of epoch 2, is forgiving of errors earlier in the epoch (threshold crossing with the wrong outcome) which might be present with larger thresholds. The results therefore reflect a tension between these conflicting features.

Further insights into the decision process with non-stationary inputs are supplied in figure 2*d*–*f*, *g*–*i*, which show (for each of the window options) the probabilities of making the wrong decision and no decision, respectively. Except at very small Δ*μ* and threshold, the un-windowed MSPRT makes an incorrect decision almost at chance for most of the parameter space. By contrast, the errors of performance for the short (300 ms) and long (700 ms) windows MSPRT are mainly a failure to reach a decision (figure 2*g*,*h*), which is not surprising given its limited time to do so.

There is some benefit for the 700 ms window from large thresholds with hard problems (small Δ*μ*). However, in the main, for the larger window size, the performance is fairly independent of threshold (up to values close to the zero-evidence bound). Thus, across both small and large windows, moderately large thresholds are preferred. Further, increasing the threshold will increase both correct and incorrect selection.

For all MSPRT variants, the tendency to no-selection (figure 2*g*–*i*) is most pronounced for hard problems and small thresholds because both conditions demand longer accumulation times. This is true even for the non-windowed case, which may require longer than 2000 ms (the experiment time).

In sum, windowing appears to confer an advantage for the MSPRT in non-stationary environments by allowing prior, irrelevant information to be discarded. For windows of smaller duration, the counter-intuitive benefits (with the current performance criterion) of somewhat larger thresholds (but below the zero-evidence limit) are explained by the limited amount of evidence available with small windows—such thresholds avoid the impasse of no selection. For larger windows (comparable with the characteristic time-scale of the stimulus) performance is fairly insensitive to the threshold but, where there is dependence, larger values (but removed from the zero-evidence limit) are preferred for the same reasons.

### 3.2. Problem-dependent windowing improves performance

Consider the performance of the windowed MSPRTs in figure 2. Both the long (700 ms) and short (300 ms) duration windows show significantly better performance than those obtained using the original MSPRT, although the longer window performs better, overall, than its shorter counterpart. Thus, conceiving of the original MSPRT as using an effectively ‘infinite’ window, these results suggest a modal progression of behaviour with window size in which there is a preferred size for best performance. Further, the preferred size is related to the epoch size (here 1000 ms). Thus longer/shorter epochs would give rise to longer/shorter preferred window size. However, selecting a window size based on the epoch duration is not a practical proposition for an autonomous agent who has no knowledge of the timing of the input transitions.

An alternative approach to selecting window size is indicated by examining the performance ‘landscape’ over the problem space. Thus, for ‘easy’ problems (with large Δ*μ*), the shorter (300 ms) window performs almost as well as the longer (700 ms). For harder problems (with small Δ*μ*), there is significant benefit in using windows longer than 300 ms. We might therefore ask the question: what is the *adequate* window duration, for each degree of problem difficulty (here, determined by Δ*μ*) that does not result in a large drop in performance? It is more realistic to suppose that an agent could estimate problem difficulty by, perhaps, separately sampling the inputs over a short, fixed window to estimate their means (we have, indeed, investigated this possibility and it does appear viable).

Based on the observations above, we assume that the adequate window size scales with the decision time for a stationary problem with three inputs. To investigate this, we therefore used a model with an un-windowed MSPRT with three, constant-mean input channels. The two ‘losing’ channels had mean *μ*^−^ and the winning channel *μ*^+^, as defined in Material and methods. The standard deviation of both channels was 0.33, and Δ*μ* was varied in the interval [0.6,2.6]. Although we did not conduct an exhaustive search, good results in the non-stationary experiments were subsequently obtained for a 15% error rate with the un-windowed MSPRT. For consistency, we also used the threshold for this error rate (0.05) in the subsequent, non-stationary input experiments. Furthermore, using a 5% error rate in the power law determination resulted in only a 1% decrease in mean reward (compared with that for 15% rate).

**Figure 3.**
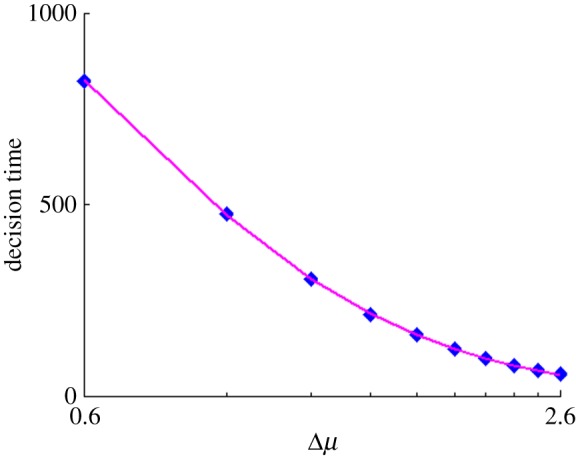
Figure depicts decision time versus decision difficulty. Blue dots represent mean decision time against Δ*μ* (log axis) in a three choice task with stationary inputs over 1000 runs. Curve shows power law fitted to decision time.

The results, averaged over 1000 runs at each value of Δ*μ*, are shown in figure 3. The data were well fitted by a power law relating the mean decision time and Δ*μ*

3.6 $$DT(\Delta \mu )=445.7\Delta \mu^{-1.98}.$$

We then used this to determine the problem-dependent window size *T*_*w*_(Δ*μ*) by simply putting *T*_*w*_(Δ*μ*)=*DT*(Δ*μ*). This method of determining *T*_*w*_(Δ*μ*) is robust. In other experiments with *N*>3 (not reported here), we used the *N*=3 relation in (3.6) with good selection performance. Moreover, we have shown that the relations like (3.6), specifically derived with *N*>3, are approximated quite well by simply scaling that for *N*=3 in (3.6).

Thus far, our evaluation of performance has rested solely on the outcome at the end of an epoch. However, in order to better gauge performance in terms of the decision *throughout* an epoch, we introduce a metric whose value is governed by the *duration* of a correct decision. Thus, at each timestep *i* within an epoch, we set a Boolean variable *r*_*i*_ to 1 if the correct channel (and only this channel) had been selected, and −1 if an incorrect channel was selected. The metric *R*_*j*_ was then defined as the sum of all such values over the epoch *j*.

3.7 $$R_{j}=\sum_{i\in J}r_{i},$$

where *J* is the set of timesteps in epoch *j*. This captures the notion that early (and sustained) correct decisions are better than late, or incorrect ones (with reference to equation (3.5), the correct outcome in epochs 1 and 2 are selection of channels 1 and 2, respectively).

Experiments with the problem-dependent window were conducted over a range of differences in mean Δ*μ*=*μ*^+^−*μ*^−^, and standard deviation *σ*, common to both the inputs. We therefore refer to *R*_*j*_(*σ*,Δ*μ*) with respect to a particular parameter pair. It is also useful to refer to the mean of *R*_*j*_ over a set of experiments. Thus, if *L*,*M* are the experimental sets of *σ* and Δ*μ* respectively, we define the mean reward in epoch *j*

3.8 $${\bar{R}}_{j}={\left\langle R_{J}(\sigma ,\Delta \mu )\right\rangle }_{L,M}.$$

As suggested by the notation, the metric in (3.7) might be interpreted as some kind of cumulative ‘reward’ for correct decision-making—early, correct decisions lead to larger values of accumulated reward—and we will therefore refer to it in this way. Note that the symmetry of *R* about zero penalizes performance at chance which will yield expected reward values of zero.

**Figure 4.**
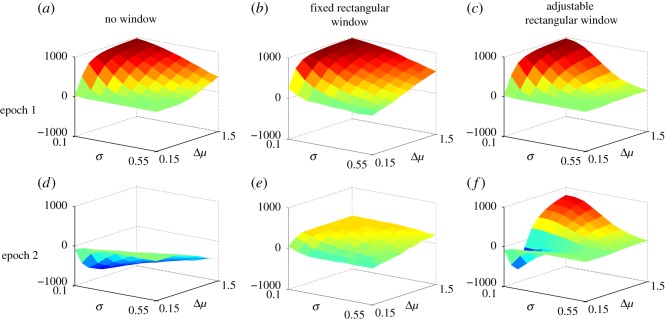
Cumulative reward in the experiment of equation (3.5); average of 1000 runs. Panels (*a*–*c*), (*d*–*f*) for *R*_1_,*R*_2_ (epochs 1, 2), respectively. Panels (*a* and *d*), (*b* and *e*), (*c* and *f*) show the case for: no window, fixed (700 ms) window and problem-dependent window (determined by (3.6)), respectively.

In the following experiments the threshold was fixed at 0.05, the value used to obtain equation (3.6). The parameter sets *L*,*M* (defining variations in *σ*,Δ*μ*) comprised the 10, equi-spaced values in the closed intervals [0.1,0.55], [0.15,1.5], respectively, and each pair of parameters was used in 1000 trials. Experiments were done with a fixed window of 700 ms, no window and the variable window defined by (3.6); the results are shown in figure 4.

For epoch 1 (figure 4*a*–*c*), there is little difference in performance (reward) across window variation. However, the results in the first epoch are not typical of the non-stationary decision process because, during this time, there is no prior evidence to contaminate the decision (and because both the fixed and un-windowed mechanisms accumulate more evidence in epoch 1, they are able to outperform the variable window mechanism).

By contrast, for epoch 2, the reward with fixed or problem-dependent (adjustable) window is significantly higher than that with no window. Unlike epoch 1, in epoch 2, a prior decision has to be reversed and prior evidence is an impediment to making the new, correct choice, thereby favouring the shorter problem-dependent windows. The problem-dependent and fixed (700 ms) windows produce similar mean rewards of 146 and 156, respectively. However, the performance of the problem-dependent window is better over large parts of the ‘easier’ problem space.

**Figure 5.**
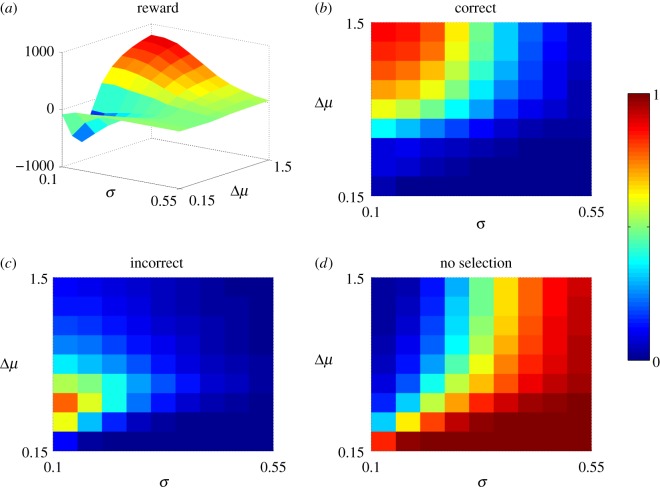
(*a*–*d*) Behaviour of adjustable rectangular window in epoch 2, from figure 4*f*. Panel (*b*) depicts the normalized amount of correct selection, panel (*c*) depicts incorrect selection, and panel (*d*) depicts no selection. Range of values shown on the right pertains to decision figures (not to reward figure).

In order to further understand the behaviour with the problem-dependent window, figure 5 shows a dissection of the behaviour in epoch 2 for the case shown in figure 4*f* into each decision possibility at the end of the epoch. Thus, in the ‘correct decision’, channel 2 is selected at the end of epoch 1, in an ‘incorrect decision’ it is channel 1 or 3, and ‘no selection’ implies no channel has crossed threshold. Multiple selection was omitted because it did not occur in significant numbers. It is clear that the failure to reach a reward maximum is largely due to failure to make a selection of any kind.

#### 3.2.1. Robustness under moderate change in gain

Thus far the accumulated evidence has made use of a windowed sum of data samples *x*(*t*), multiplied by a gain *g*, where *g*=(*μ*^+^−*μ*^−^)/*σ*^2^; see equation (2.3). The evaluation of this gain would seem to require the mechanism has knowledge of its own inputs (means and standard deviation). However, in [18] it was shown that the performance of the basic (window-free) MSPRT mechanism was independent of the gain, as long as it was larger than the nominal value given here. We therefore sought to find the effect of changing the gain on performance in the windowed mechanism described here. This was done by taking *g* defined above and multiplying by a factor *k*, with *k*=0.5,1,5,10,20.

**Figure 6.**
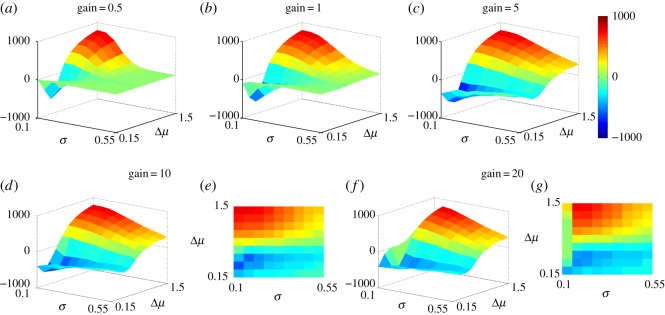
Effect of gain on performance with the adaptive window. Cumulative reward *R* in epoch 2 using the protocol in equation (3.5) shows the original result with gain *g*=(*μ*^+^−*μ*^−^)/*σ*^2^. Panels (*a* and *c*) are for the case when this gain is multiplied by 0.5 and 5, respectively. Panels (*d* and *e*) and (*f* and *g*) are for the case when the gain is multiplied by 10 and 20, respectively, both in three-dimensional form and as a two-dimensional contour plot, to highlight features otherwise masked by the view in three dimensions (which is consistent with the other panels).

The results for epoch 2 are shown in figure 6. In addition, the mean rewards for each *k* (in order of increasing *k*) were 102,146,199,176,129, respectively. Thus, when the gain is below nominal, the performance is compromised. For moderate increases in gain, *k*=5,10, there is an improvement in average reward, and inspection of figure 6 shows this to occur mainly for large Δ*μ* and *σ*.

This is significant because one limitation of our adaptive window approach is that, while it tries to accommodate the type of decision at hand (by taking into account the variation of Δ*μ*), it does not take into consideration how long we have to make a decision before a new, more salient option is presented (length of window can become larger than epoch size for very difficult decisions). This can therefore result in sub-optimal window sizes which reduce the overall level of reward. Moderate increases in gain appear to overcome this to some extent since they force more rapid accumulation, thereby preventing non-selection or reducing the delay in selecting, which can be preferred in cases where the overall reward is time dependent. This also accounts for the decrease in reward for lower Δ*μ* and higher *σ*, when the gain is increased above 1. In that case, while our mechanism takes into account the variation in problem difficulty, it does so only with reference to Δ*μ*. As such, when Δ*μ* and *σ* are of similar magnitude, the window size becomes sub-optimal. This is enhanced by the higher gain, which promotes quicker selection and therefore more erroneous decisions.

#### 3.2.2. Exponential window geometry

Thus far, we have considered the moving accumulation window to have a rectangular shape. Conceptually, this is straightforward to understand and it would appear to make best use of the evidence available since all such is equally weighted. However, from an implementation perspective it has the drawback that it is necessary to keep a record of all evidence samples over a period *T*_*w*_, in order to know what contribution is to be removed from the tail of the window at each timestep. While this is not significant in a machine learning context it may have implications in future attempts to couch the mechanism in biologically plausible form.

To address this issue, consider a non-rectangular window defined by the exponential function

3.9 $$w(t)=\left\{ \begin{array}{ll} g\exp(-\lambda t), & \text{if }t\geq 0\\ 0, & \text{if }t<0 \end{array}\right.,$$

where λ=1/*τ*_*w*_ and *τ*_*w*_ is a characteristic time constant measured in timesteps, and *g* is given in (2.3). Then convolving this with the data *x*(*t*) (channel suffix suppressed), and using the limit on non-zero *w*(*t*), we obtain the equivalent of (3.4)

3.10 $$y(T)=g\sum_{t=1}^{T}x(t)\exp[-\lambda (T-t)].$$

At the next timestep,

3.11 $$\begin{array}{rl} y(T+1) & =g\sum_{t=1}^{T+1}x(t)\exp[-\lambda (T+1-t)]\\ & =\exp(-\lambda )\left\{g\sum_{t=1}^{T}x(t)\exp[-\lambda (T-t)]+gx(T+1)\exp(\lambda )\right\}\\ & =\exp(-\lambda )y(T)+gx(T+1). \end{array}$$

Assuming many timesteps for the exponential to decay significantly, *τ*_*w*_≫1 or λ≪1, so that exp⁡(−λ)≈1−λ. Using this in (3.11),

3.12 y(T+1)=(1−λ)y(T)+gx(T+1).

Thus, there is a very simple, memory-efficient update rule for accumulating evidence with the exponential window: reduce or ‘decay’ the current accumulated evidence *y*(*T*) by a factor 1−λ and add in the new (gain-weighted) evidence sample.

To fix *τ*_*w*_ (and hence λ) in a way which enabled meaningful comparison with previous results, we chose to use a problem-specific adaptive window and obtain λ using the power law in (3.6). Thus, we set

3.13 $$\lambda (\Delta \mu )=\frac{1}{DT(\Delta \mu )}.$$

**Figure 7.**
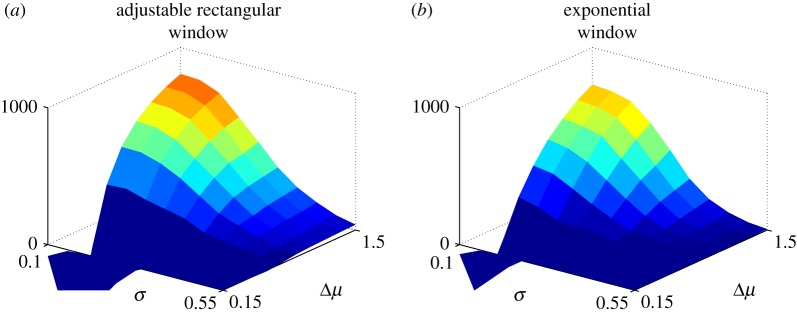
Comparison of cumulative reward *R* in epoch 2 from problem specific windows with rectangular and exponential form using the protocol in equation (3.5) (each averaged over 1000 runs). Panel (*a*) shows results for the rectangular window; panel (*b*) shows results for the exponential window (repeated here from figure 5 for ease of comparison).

Figure 7 shows the resulting reward in epoch 2 (typical of decisions in general non-stationary situations) using the protocol in equation (3.5). For ease of comparison, corresponding results for the adaptive rectangular window are show alongside. The mean reward ${\bar{R}}_{2}$ for the rectangular and exponential windows was 146 and 110, respectively.

In order to test whether choices for λ(Δ*μ*) other than that (3.13) could yield better results, we investigated the effect of introduced a scaling term *n* so that λ=*n*/*DT*, with 0.5≤*n*≤5. The values of ${\bar{R}}_{2}$ for *n* in a sequence {0.25,0.5,1,2,5} were {−102,27,110,128,89}. These results show a modal behaviour of performance against scaling, and that there is no substantive loss in performance by adopting the nominal value of λ in (3.13) with *n*=1.

Mechanisms with fixed λ, for a variety of values, were also tested but systematically under-performed compared with those with variable window size. This is in line with similar results for the rectangular window.

## 4. Discussion

To work effectively in a continuous-time manner with non-stationary inputs, a decision mechanism must have some way to reset or ‘forget’ accumulated evidence which is no longer relevant. We sought to address this issue in the context of MSPRT-based algorithms which, hitherto, to the best of our knowledge, have not been equipped in this way. Tsetsos *et al.* [23] conducted a comparative study of several decision mechanisms in non-stationary input environments. The target mechanisms were the race model [28], the drift diffusion model [29] and the leaky competing accumulator model (LCA) [26]. The authors found that, with correlated evidence, the LCA was best able to explain results from comparable experiments with human subjects. The authors did not, however, test the MSPRT model in such settings.

Through the introduction of a rectangular integration window with a transparent decision boundary, we were able to make an MSPRT-based algorithm select and de-select options appropriately based on their relative magnitude. We also showed that the use of such a window yields better performance than the original un-windowed algorithm, across the range of parameters tested.

Performance depends on the size of the window which is related to epoch duration—a parameter which is unknowable by an autonomous agent. Thus, with the aim of establishing principles for use in such a context, we investigated the use of a window whose duration varied according to problem difficulty, and which could, in principle, be estimated dynamically. Performance held up well with such a mechanism over a large range of the problem space. Further, results were robust with respect to changes in the parameters of the mechanism for determining window size (power laws fitted to the outcome of un-windowed experiments).

Rectangular windows are, conceptually, the simplest possibility to evaluate, as they imply the use of a uniformly weighted set of recent evidence. Thus, they present a natural benchmark against which other windowing methods may be compared. However, we did go on to explore an alternative to the rectangular window, which used less memory resources. This mechanism added new evidence at each timestep while exponentially decaying older evidence. Even though its performance was inferior to that of the rectangular window, it still presents a viable alternative to it, in cases where memory is limited, and may form a basis for biologically plausible implementations of our method.

In regards to this approach, our attention was drawn during review to work of which we were not previously aware [30]. The authors implemented a nonlinear stochastic model of evidence accumulation which uses exponential discounting as a means of discarding outdated evidence. In their implementation, however, the evidence discount rate is dependent on the frequency of environmental changes and assumes that those are always known to the decision maker. We believe this to be fundamentally different from our own implementation of the exponential window, with the only commonality being the geometry of the discounting window. In our implementation, λ is calculated through the use of a power law which depicts decision time for a given number of competing channels and for a particular Δ*μ*, which we discovered empirically.

As already noted, one candidate for use in non-stationary input situations is the LCA [23]. However, this has more free parameters than our algorithm and so would appear to be harder to implement in an autonomous agent. Further, in terms of optimality, Bogacz *et al.* [31] showed that, even though the LCA is straightforward to implement for *N*>2 alternatives by setting the inhibition and decay to the same high value, for *N*=2 it requires further manipulations and parameter search in order to achieve the same statistical optimality of the SPRT mechanism. The LCA does achieve optimal performance in the so-called ‘interrogation paradigm’, in which a decision is forced if none is reached within a certain amount of time. However, because we are interested in using a transparent boundary for decision, these results do not directly compare with those presented here.

This paper has sought to address a gap in the MSPRT literature by presenting a principled way to accumulate evidence in a continuous, non-stationary setting, allowing evidence to be discarded dynamically without the need for a reset to the mechanism. Our problem-dependent windowing methods (both rectangular and exponential) require knowledge of the smallest difference between means of input signals and, while preliminary work suggests this may also be estimated in an ‘online’ manner, future work is required to fully evaluate this approach.

## Data accessibility

Data are available at Dryad repository: .

## References

[1] Bogacz R, Brown E, Moehlis J, Holmes P, Cohen JD 2006 The physics of optimal decision making: a formal analysis of models of performance in two-alternative forced-choice tasks. *Psychol. Rev.* 113, 700–765. (doi:10.1037/0033-295X.113.4.700) 17014301 10.1037/0033-295X.113.4.700

[2] Usher M, Tsetsos K, Yu EC, Lagnado Da 2013 Dynamics of decision-making: from evidence accumulation to preference and belief. *Front. Psychol.* 4, 758 (doi:10.3389/fpsyg.2013.00758) 24151480 10.3389/fpsyg.2013.00758 PMC3798759

[3] Gold JI, Shadlen MN 2007 The neural basis of decision making. *Annu. Rev. Neurosci.* 30, 535–574. (doi:10.1146/annurev.neuro.29.051605.113038) 17600525 10.1146/annurev.neuro.29.051605.113038

[4] Heekeren HR, Marrett S, Ungerleider LG 2008 The neural systems that mediate human perceptual decision making. *Nat. Rev. Neurosci.* 9, 467–479. (doi:10.1038/nrn2374) 18464792 10.1038/nrn2374

[5] Churchland AK, Ditterich J 2012 New advances in understanding decisions among multiple alternatives. *Curr. Opin. Neurobiol.* 22, 920–926. (doi:10.1016/j.conb.2012.04.009) 22554881 10.1016/j.conb.2012.04.009 PMC3422607

[6] Smith PL, Vickers D 1988 The accumulator model of two-choice discrimination. *J. Math. Psychol.* 32, 135–168. (doi:10.1016/0022-2496(88)90043-0)

[7] Ratcliff R, Rouder JN 1998 Modeling response times for two-choice decisions. *Psychol. Sci.* 9, 347–356. (doi:10.1111/1467-9280.00067)

[8] Cho RY, Nystrom LE, Brown ET, Jones AD, Braver TS, Holmes PJ, Cohen JD 2002 Mechanisms underlying dependencies of performance on stimulus history in a two-alternative forced-choice task. *Cogn. Affective Behav. Neurosci.* 2, 283–299. (doi:10.3758/CABN.2.4.283) 10.3758/cabn.2.4.283 12641174

[9] Ratcliff R, Mckoon G 2008 The diffusion decision model: theory and data for two-choice decision tasks. *Neural Comput.* 922, 873–922. (doi:10.1162/neco.2008.12-06-420) 10.1162/neco.2008.12-06-420 PMC2474742 18085991

[10] Wald A, Wolfowitz J 1948 Optimum character of the sequential probability ratio test. *Ann. Math. Stat.* 19, 326–339. (doi:10.1214/aoms/1177730197)

[11] Churchland AK, Kiani R, Shadlen MN 2008 Decision-making with multiple alternatives. *Nat. Neurosci.* 11, 693–702. (doi:10.1038/nn.2123) 18488024 10.1038/nn.2123 PMC2453226

[12] McMillen T, Holmes P 2005 The dynamics of choice among multiple alternatives. *J. Math. Psychol.* 50, 30–57. (doi:10.1016/j.jmp.2005.10.003)

[13] Ditterich J 2010 A comparison between mechanisms of multi-alternative perceptual decision making: ability to explain human behavior, predictions for neurophysiology, and relationship with decision theory. *Front. Neurosci.* 4, 184 (doi:10.3389/fnins.2010.00184) 21152262 10.3389/fnins.2010.00184 PMC2999395

[14] Krajbich I, Rangel A 2011 Multialternative drift-diffusion model predicts the relationship between visual fixations and choice in value-based decisions. *Proc. Natl Acad. Sci. USA* 108, 13852–13857. (doi:10.1073/pnas.1101328108) 21808009 10.1073/pnas.1101328108 PMC3158210

[15] Baum CW, Veeravalli VV 1994 A sequential procedure for multihypothesis testing. *IEEE Trans. Info. Theory* 40, 1994–2007. (doi:10.1109/18.340472)

[16] Dragalin VP, Tartakovsky AG, Veeravalli VV 1999 Multihypothesis sequential probability ratio tests—part I : asymptotic optimality. *IEEE Trans. Info. Theory* 45, 2448–2461. (doi:10.1109/18.796383)

[17] Dragalin VP, Tartakovsky AG, Veeravalli VV 2000 Multihypothesis sequential probability ratio tests—part II: accurate asymptotic expansions for the expected sample size. *IEEE Trans. Info. Theory* 46, 1366–1383. (doi:10.1109/18.850677)

[18] Bogacz R, Gurney K 2007 The basal ganglia and cortex implement optimal decision between alternative actions. *Neural Comput.* 19, 442–477. (doi:10.1162/neco.2007.19.2.442) 17206871 10.1162/neco.2007.19.2.442

[19] Lepora N, Gurney K 2012 The basal ganglia optimize decision making over general perceptual hypotheses. *Neural Comput.* 11, 2924–2945. (doi:10.1162/NECO_a_00360) 10.1162/NECO_a_00360 22920846

[20] Redgrave P, Prescott TJ, Gurney K 1999 The basal ganglia: a vertebrate solution to the selection problem? *Neuroscience* 89, 1009–1023. (doi:10.1016/S0306-4522(98)00319-4) 10362291 10.1016/s0306-4522(98)00319-4

[21] Ding L, Gold JI 2013 The basal ganglia’s contributions to perceptual decision making. *Neuron* 79, 640–649. (doi:10.1016/j.neuron.2013.07.042) 23972593 10.1016/j.neuron.2013.07.042 PMC3771079

[22] Ossmy O, Moran R, Pfeffer T, Tsetsos K, Usher M, Donner TH 2013 The timescale of perceptual evidence integration can be adapted to the environment. *Curr. Biol.* 23, 981–986. (doi:10.1016/j.cub.2013.04.039) 23684972 10.1016/j.cub.2013.04.039

[23] Tsetsos K, Usher M, McClelland JL 2011 Testing multi-alternative decision models with non-stationary evidence. *Front. Neurosci.* 5, 63 (doi:10.3389/fnins.2011.00063) 21603227 10.3389/fnins.2011.00063 PMC3093747

[24] Caballero JA, Lepora NF, Gurney KN 2015 Probabilistic decision making with spikes: from ISI distributions to behaviour via information gain. *PLoS ONE* 10, e0124787 (doi:10.1371/journal.pone.0124787) 25923907 10.1371/journal.pone.0124787 PMC4414410

[25] Chevalier G, Deniau JM 1990 Disinhibition as a basic process in the expression of striatal functions. *Trends Neurosci.* 13, 277–280. (doi:10.1016/0166-2236(90)90109-N) 1695403 10.1016/0166-2236(90)90109-n

[26] Usher M, McClelland JL 2001 The time course of perceptual choice: the leaky, competing accumulator model. *Psychol. Rev.* 108, 550–592. (doi:10.1037/0033-295X.108.3.550) 11488378 10.1037/0033-295x.108.3.550

[27] Vickers D 1970 Evidence for an accumulator model of psychophysical discrimination. *Ergonomics* 13, 37–58. (doi:10.1080/00140137008931117) 5416868 10.1080/00140137008931117

[28] Audley RJ, Pike AR 1965 Some alternative stochastic models of choice. *Br. J. Math. Stat. Psychol.* 18, 207–225. (doi:10.1111/j.2044-8317.1965.tb00342.x)

[29] Stone M 1960 Models for choice-reaction time. *Psychometrika* 24, 251–260. (doi:10.1007/BF02289729)

[30] Veliz-Cuba A, Kilpatrick ZP, Josić K 2015 Stochastic models of evidence accumulation in changing environments. *SIAM Rev.* 58, 264–289. (doi:10.1137/15M1028443)

[31] Bogacz R, Usher M, Zhang J, McClelland JL 2007 Extending a biologically inspired model of choice: multi-alternatives, nonlinearity and value-based multidimensional choice. *Phil. Trans. R. Soc. B* 362, 1655–1670. (doi:10.1098/rstb.2007.2059) 17428774 10.1098/rstb.2007.2059 PMC2440778

